# Salinity effects on the strength and morphological indices of soft marine clay

**DOI:** 10.1038/s41598-022-22627-w

**Published:** 2022-10-20

**Authors:** Weijuan Geng, Wenxia Han, Jie Yin, Zhijun Lu

**Affiliations:** 1 Department of Civil Engineering, Faculty of Civil Engineering and Mechanics, Jiangsu University, Zhenjiang, 212013 China; 2 Key Laboratory of Ministry of Education for Geomechanics and Embankment Engineering, Hohai University, Nanjing, 210096 China; 3 Department of Civil Engineering, Jiangsu Urban and Rural Construction College, Changzhou, 213147 China

**Keywords:** Civil engineering, Environmental sciences

## Abstract

This study evaluates the strength behaviors and morphological characteristics of Lianyungang marine clay under the effect of porewater salinity. Soil at higher salinity was found to have increased internal friction angle and undrained shear strength. The difference in undrained shear strength enlarges as the confining pressure increases. Different stress paths were exhibited with soil at different salinities. Soil morphology analysis including scanning electron microscopy (SEM) and Image-Pro Plus (IPP) were employed to investigate the underlying mechanism of the enhanced strength behaviors of soft marine clay with increased salinity. Aggregated soil fabric was observed at higher salinity and contributed to enhanced strength. The results demonstrate that the aggregated soil structure is the primary mechanism responding to the enhanced strength behavior of marine clay under relatively high salinity (6%). Quantitative relationships were developed between the strength parameters and morphological characteristics of soil, i.e., area of particles, roundness of particles, area of pores, pore orientation, and fractal dimension of pore distribution, in the forms of empirical equations, and are expected to serve as the references for prediction in undrained behaviors of soft marine clays with known soil index.

## Introduction

Quaternary sediments such as soft marine clay are widely distributed in the coastal regions of many countries, i.e., the United States, China, Thailand, Korea, Japan, Malaysia, Singapore, etc., forming by gradually receded coastline under the invasion of seawater^1–7^. With the rapid development of infrastructure in coastal areas, a large amount of in-situ marine clay needs to be reclaimed as construction sites; however, these lands could not satisfy the ground requirements due to the characteristics of high plasticity, high compressibility, low strength, low hydraulic conductivity, and low bearing capacity^8–11^. Meanwhile, freshwater from rainfall and unfrozen glaciers reduces the salinity of porewater in marine soils, causing problems in large and/or differential settlements and bearing capacity failures of foundation^12–17^. Therefore, studying the mechanical properties of soft marine clays would be beneficial in avoiding adverse geological problems, i.e., ground subsidence and landslide, and providing robust design parameters for related constructions.

Strengths are the primary mechanical properties of soft soil, which determine the deformation and stability of foundations, especially in marine areas. Researches have been conducted on the effects of chemical composition and salt concentration of pore water on the mechanical properties of soil, including compressibility (e.g.,^8,18–20^) and shear strength (e.g.,^21–27^). The results show that pore water has a significant effect on the mechanical properties of soft marine soil^28^. For example, Sridharan and Prakash^29^ found that the undrained shear strength of kaolinite was with an opposite developing trend to that of montmorillonite under different physiochemical environments. Maio et al.^30^ conducted direct shear tests with saturated montmorillonite and kaolinite in sodium chloride (NaCl) solutions and concluded that the shear strength of montmorillonite increased with increasing salinity of pore water, whereas that of kaolinite would not change much with the salinity. It attributed the increased shear strength to the thinned thickness of the diffused double layer (DDL) of montmorillonite under a high salt concentration of porewater, where the friction between soil particles was strengthened. Spagnoli et al.^28^ stated that the shear strengths of kaolinite and illite would not be influenced much by the solutions of different electric conductivity (EC), ionic strength (*I*), and pH; however, that of montmorillonite was highly sensitive to the properties (e.g., EC, *I* and pH) of salt solutions. Though a number of soil additives, including lime^31^, cement^32,33^, biomass silica^34^, and waste materials (e.g.,^11,34–37^), have been studied to improve the strength of marine clay, the mechanism of the porewater salinity effect on macroscopic engineering behaviors of soft marine clay is yet to develop in the context of coastal land development. It needs to point out that the existing research showed inconsistent conclusions on the strength characteristics of soft soil subjected to salinity change. Some concluded that the salinity exerted a negative impact on the strength development of soft soil (e.g.,^38,39^, etc.), whereas some stated that the increase of salinity led to higher viscous resistance and increased strength (e.g.,^40,41^, etc.). Therefore, there is a need to investigate the salinity effect on the strength behaviors of a natural soft marine clay—Lianyungang clay, where the region is in urgent demand for the reclamation purpose as ground soil, and the corresponding changes of its morphological indices.

The objective of this study is to investigate the effect of porewater salinity on the strength of a natural soft marine clay obtained from Lianyungang Port and to interpret the relationship between strength behaviors and morphological indices to serve as potential screening tools for assessing the strength of in-situ marine clay in Lianyungang region. Isotropic consolidated undrained triaxial compression tests were conducted on the remolded soft marine clay of different porewater salinity using NaCl solution at targeted concentrations. Scanning electron microscopy (SEM) imaging analysis was used to visualize the microscopic structure changes of marine clay. Image-Pro Plus (IPP) program was employed to analyze relationships between strength parameters and soil morphological characteristics. Quantitative relationships between the strength parameters and the morphological indices were developed and could serve as potential references for predicting undrained behaviors of soft marine clays with known soil index. Additionally, the results achieved in this study enhance the understanding of the effects of porewater salinity on the macroscopic engineering properties of soft marine clay.

## Materials and methods

### Soft marine clay

**Figure 1:**
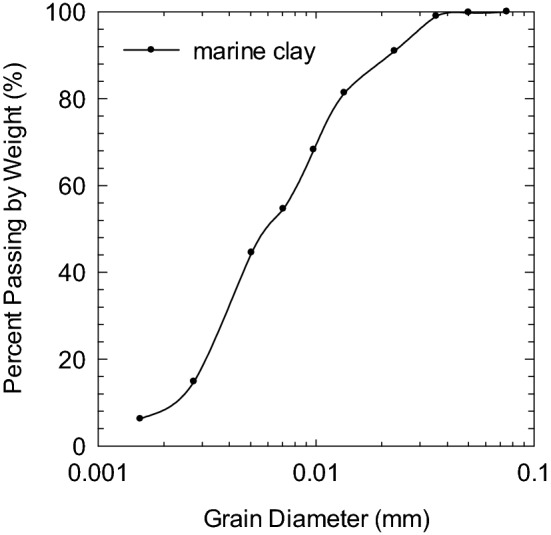
Grain size distribution of marine clay.

**Figure 2:**
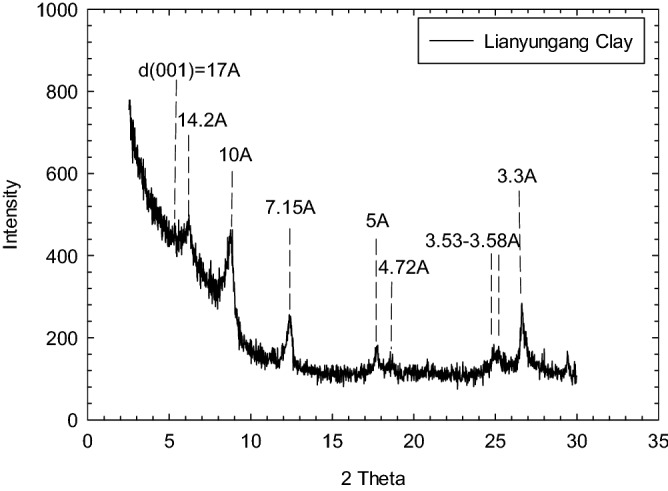
X-ray diffraction (XRD) scan pattern of Lianyungang Clay.

Soft marine clay was collected from a construction site at Lianyungang Port, Jiangsu Province, China. The port is at the northern toe of Houyuntai Mountain by the shoreline of the Yellow Sea, located at the eastern end of the narrow area between Houyuntai Mountain and Dongxilian Island. The underwater terrain is slightly inclined to the east and is partially uplifted as a result of reclamation by pumping fillings, where the ground surface elevation is at 1.40–2.00 m. The construction site is located in the middle section of the northwestern wing of the Lianyungang-Fushan inverted tilt. The Quaternary loose deposits are presented in the entire site investigation range. The base rock belongs to pre-Sinian metamorphic rock-gneiss, where the rock formation is northeastward leaning southeast, defined as a monoclinic structure. Intermittent up-down neotectonic movement was exhibited in this area, causing marine and terrestrial strata to appear within the investigated depth alternately. A saturated soft plastic clay layer was formed within the sea regression period and is widely distributed in the investigated site at shallow depths. The average thickness of the soft clay layer is 8.7 m, sandwiched by a thin crust layer (~ 0.5 m) and a sub sand/stiff clay layer^42^. Soil used in this study was collected from a block sample at a depth of 2.5 m below the ground surface (soft clay layer), where the average groundwater table was located 1.5 m below the ground surface. The liquid limit (LL) and plastic limit (PL) of soil were 56.4% and 28.6%, respectively, with a plasticity index (PI) of 27.8. Table 1 summarizes the physical properties of natural soil per ASTM D422-63(2007). The particle size analysis is shown in Fig. 1, where more than 50% of grains with particle sizes are finer than 0.075 mm. Soil is classified as clay of high plasticity (CH) according to the United Soil Classification System (USCS). Quantitative X-ray diffraction (XRD) analysis on the mineralogy of the soil showed the soft marine clay to consist of 59% montmorillonite/illite, 31% illite, 5% kaolinite, and 4% chlorite (Table 2), as shown in Fig. 2. Sodium chloride was detected as the primary salt content of seawater at the site, where the chloride concentration was measured at 16,248–16,294 mg/L.

**Table 1:** Physical properties of soft marine clay.

Water content	Unit weight	Specific gravity	Liquid limit	Plastic limit	Plasticity index
%	kN/m^3^	–	%	%	–
48.2–49.5	17.8	2.7	56.4	28.6	27.8

**Table 2:** Mineralogy of soft marine clay used in this study.

Mineral	Montmorillonite/Illite	Illite	Kaolinite	Chlorite	Ordered mixed layer
Relative abundance (%)	59	31	5	4	1

### Sample preparation and testing

#### Triaxial test

Sodium chloride (NaCl) solutions are made at a series of concentrations as to the porewater of soil for triaxial tests in this study. NaCl solutions are prepared by dissolving reagent grade NaCl (content of NaCl is greater than 99.5%) in deionized (DI) water (EC ≤ 0.1 μs/cm). The solution concentrations (S) were calculated as the ratio between the dry mass of solvent to the mass of solution at 2%, 4%, and 6%. Soil obtained from the site was soaked in DI water for 7 days to wash away the dissolved ions and dried in an oven at a relatively low temperature (~ 60 °C). The dried soil was then passed through the 2 mm sieve and mixed with DI water and salt solutions at the targeted concentration (e.g., 2%, 4%, 6%) to have the soil slurry with a water content of 1.25 times of liquid limit (*w*% at 70.5%). The soil slurries were mixed evenly for 10 min by a mechanical mixer, then put in a sealed box for 24 h to prevent water evaporation at room temperature ($20\pm 5 ^\circ \mathrm{C}$). Soil slurries were mixed again after curing prior to pouring slowly into a cylindrical PVC mold with an inner diameter of 64 mm and a height of 25 cm. The pouring was processed in three stages, where the molds were tapped to remove entrapped air in the mixture for each stage. Then the soil in PVC mold was consolidated with drainage to 25 kPa by incremented consolidation loads (load increment ratio of unity) in an oedometer, where each increment load was applied for 24 h. The remolded soil sample was obtained from the PVC tube and trimmed to a cylinder specimen with a diameter of 39.1 mm and a height of 80 mm. The water content of each specimen was measured by then as the water content of consolidation.

Triaxial tests were conducted using a TKA-TTS(-S) fully automatic triaxial instrument (Nanjing TKA Technology Co., Ltd). The remolded soil specimens prepared at different salinity were pre-consolidated under a pressure of 25 kPa with de-aired water and back-pressure (200 kPa) saturated until a B value of at least 0.95 was attained. Then testing specimens were isotopically consolidated under the confining pressure, which was taken as the mean effective stress ${\mathrm{p}}^{{{\prime}}}$, employed at 50 kPa, 100 kPa, and 150 kPa. After consolidation, the soil specimens were sheared in undrained condition at the rate of 0.073 mm/min (0.09% per min in axial strain). The tests were terminated when the axial strain of 20% was reached according to ASTM D4767-11(2020). Water content was then measured and correlated by Eq. (1) as follows:

1 $$w=\frac{{\mathrm{m}}_{\mathrm{w}}}{{\mathrm{m}}_{\mathrm{s}}}\times 100=\frac{{\mathrm{m}}_{\mathrm{w}}}{{\mathrm{m}}_{\mathrm{d}}-{\mathrm{m}}_{\mathrm{sa}}}\times 100$$

where *w* is the correlated water content (%), m_w_, m_d_, m_s_, m_sa_ are the masses (g) of water, solids (soil particles and NaCl crystals), soil particles, and dissolved salts, respectively. The initial index properties of soil specimen were shown in Table 3.

**Table 3:** Initial index properties of soil specimens.

Salinity	Water content after consolidation	Wet density	Initial void ratio	Saturation
%	%	g/cm^3^	–	%
0	57.21	1.631	1.612	96.15
2	52.23	1.673	1.467	96.51
4	49.05	1.685	1.395	95.28
6	47.85	1.706	1.349	96.12

#### Scanning electron microscopy (SEM)

Image analysis was conducted on soil specimens at different salinities obtained from triaxial tests. Prior to image analysis, soil samples were back pressure saturated and isotopically consolidated (confining pressure at 100 kPa). Soil samples obtained from consolidation were cautiously cut into thin specimens (~ 1 cm × 1 cm) in the center of samples (core part along the horizontal orientation) using a razor knife, mounted on holders using carbon tape. SEM images of the cut surfaces were obtained by a Hitachi S-3400 N SEM using a 3-keV electron beam and a conventional secondary electron detector.

#### Image-Pro Plus Program (IPP)

Images from SEM were imported into the IPP program (Media Cybernetics Corporation, USA) and converted to binary images, where soil particles and pores were represented by the white regions and black regions assigned with the value of zero and unity, respectively, with 256 quantization levels. The pixel classification algorithm was adopted to identify segment objects and regions using a three-step process. The IPP program is used to probe an image with a pre-defined binary structuring element on the morphology (e.g., perimeter, area, roundness, orientation, etc.) of solids (soil particles) and voids (pores) with soil specimens of different salinities. The software automatically segments structures and prepares images by morphological filters (i.e., areas, perimeters, roundness, angles, etc.). Results were exported with quantitative information on soil morphology.

## Results and discussion

### Effect of salinity on the shear strength of soft marine clay

**Figure 3:**
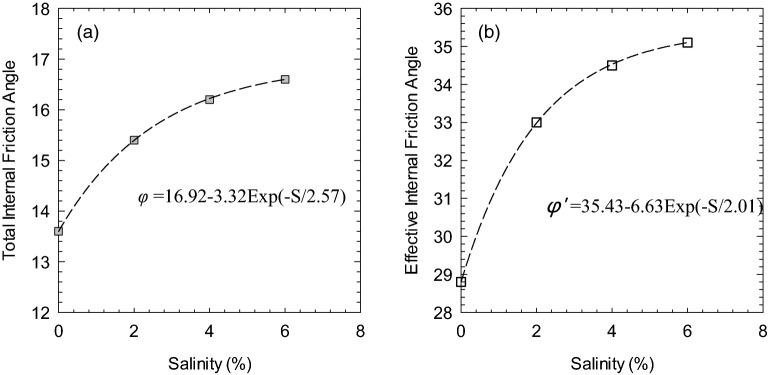
(**a**) Total internal friction angle and (**b**) Effective internal friction angle versus salinity.

The shear strength indexes were obtained by plotting the test results with soil specimens under confining pressures of 50 kPa, 100 kPa, and 150 kPa under Mohr–Coulomb failure criteria. Table 4 shows the shear strength indexes of the remolded soil specimen at each salinity. Soils are considered normally consolidated clay since the preconsolidation pressure of remolded soil is 25 kPa, which is lower than the consolidation pressure (e.g., 50 kPa, 100 kPa, and 200 kPa). The determined friction angles are plotted with salinity in Fig. 3. The total and effective internal friction angles increase as the soil salinity increases, where the maximum increase rate is reached at approximately 2% salinity. The internal friction angles can be well fitted by a trendline, where the total and effective internal friction angles are expressed in Eqs. (2) and (3).

2 $$ \varphi=16.92-3.32 {\rm Exp}(-S/2.57)$$

3 $$\varphi ^\prime=35.43-6.63 {\rm Exp}(-S/2.01) $$

where φ and φ′ are the total and effective internal friction angle, respectively, and *S* is the salinity of soil porewater.

**Table 4:** The internal friction angles of soil with different salinities.

Porewater salinity (%)	0	2	4	6
φ (°)	13.6	15.4	16.2	16.2
φ*’* (°)	28.8	33.0	34.5	35.1

**Figure 4:**
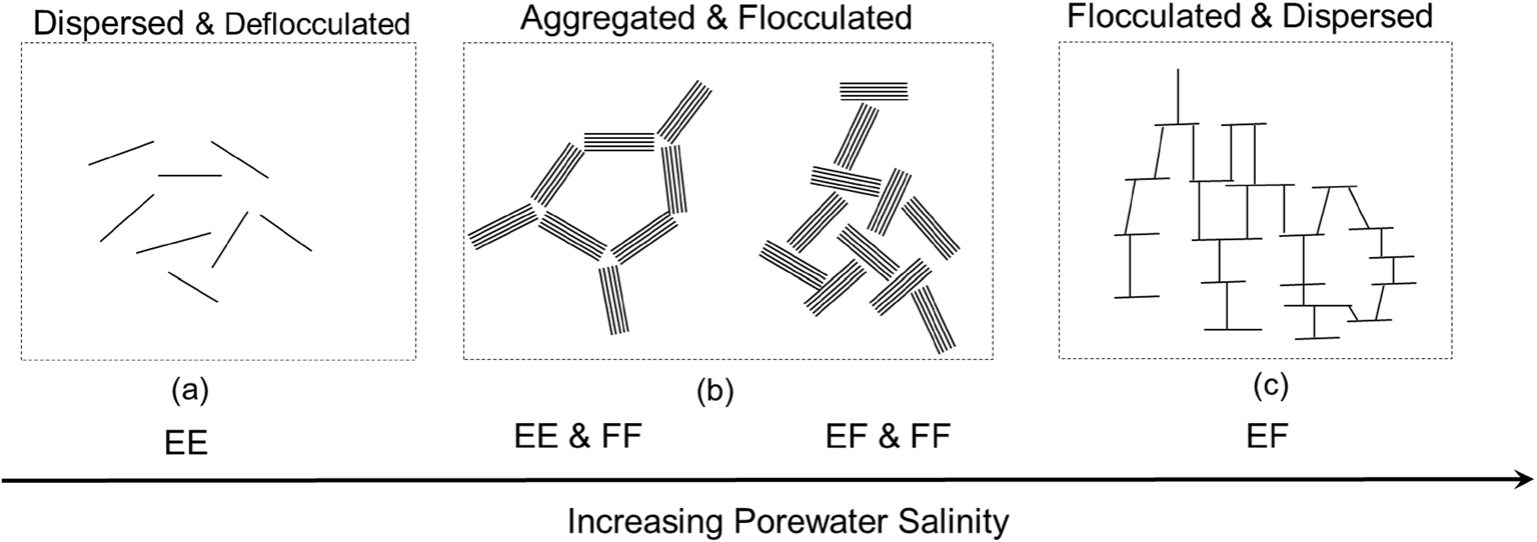
Soil structures and porewater salinity (modified after Mitchell and Soga^51^).

The higher concentration of salt ions in the pore water decreases the thickness of the diffused double layer of soil due to osmotic pressure. The thickness of the water film adsorbed on the surface of soil particles was reduced due to decreased water-binding capacity. Therefore, the double-layer shrank, resulting in an increased contact area between soil particles that impedes the relative displacement of soil particles, and presents an increased internal friction angle. Also, the structure of soil particles changes in the saline environment, which is closely related to the internal friction angle of soft soil. As shown in Fig. 4, the soil is with a dispersed and deflocculated structure as the repulsion forces between soil pellets are strong (a); as the salinity of soil porewater increases, there would be more face-to-face and edge-to-edge or edge-to-face associations between soil pellets contributing to an aggregated and flocculated soil structure, where the soil primarily contains large aggregates (b). The shearing resistance of soil would be increased as a result of a more aggregated soil structure, where the attraction force increases and the repulsion force enhances due to reduced thickness in the diffused double layer of high salinity soil^43^. Thus the ability to resistance against shearing is improved. In addition, the elastic modulus of saline soil is higher than that of normal soil. The formed soil aggregates due to saline solution alter the structure of remolded soil, causing the increased rate of increasing soil strength^44,45^. However, it needs to note that the association of soil particles would not keep increasing as the salinity continues to increase, where less face-to-face association would present and soil fabric would be dominated by edge-to-face association; thus, the soil would be with a flocculated but dispersed structure in this condition (c).

### Effect of salinity on the effective stress path of soft marine clay

**Figure 5:**
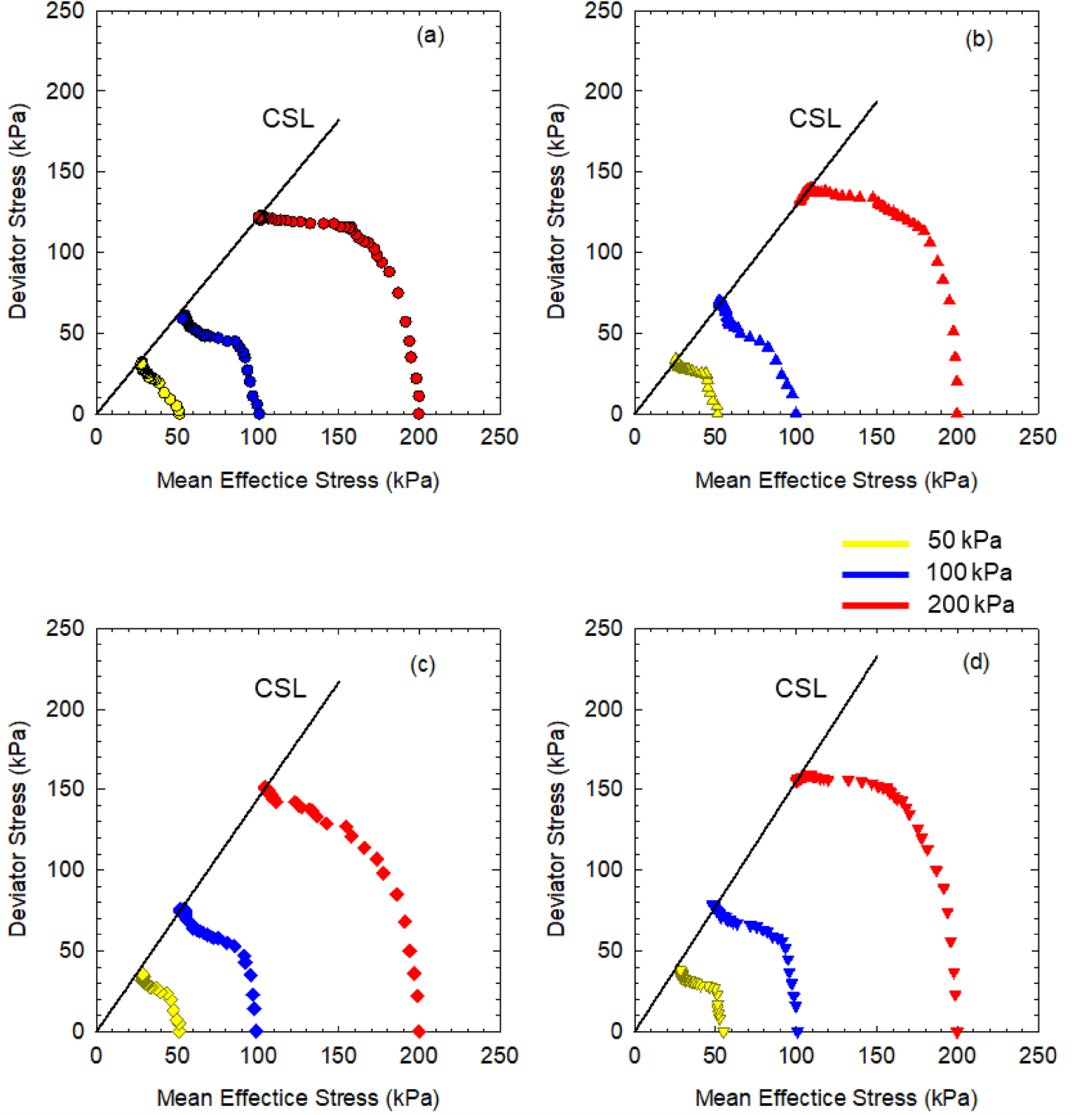
Effective stress path of soft marine clay with porewater salinity at (**a**) *S* = 0%; (**b**) *S* = 2%; (**c**) *S* = 4%; (**d**) *S* = 6%.

By Terzaghi’s Consolidation Theory, the excess pore water pressure changes with time under loading, thus the stress state changes for any point in soil. Triaxial results plotted in p-q space can describe the continuous stress change of soil during the loading process. The critical state line (CSL) defines the relationship between the mean effective stress, deviator stress, and void ratio of soil at critical state in three-dimensional space. The projected curve on p-q space is described in Eq. (4) as follows:

4 $$\mathrm{q}=\mathrm{Mp{^{\prime}}}$$

where M is the ratio of q to $\mathrm{p{^{\prime}}}$ at the critical state. Figure 5 plots the effective stress path in the p–q space of soft marine clay at different salinities. The stress paths exhibit similar behaviors with soil at different salinities since all the soil specimens are considered as normally consolidated clays. Soil specimens were under elastic deformation initially, then the stress path was towards up left nonlinearly due to the change in pore water pressure. The deviator stress reached the maximum value as the mean effective stress decreased, indicating the soil was in the critical state. The stress path would continue towards the down-left along with the CSL after the shear failure.

**Figure 6:**
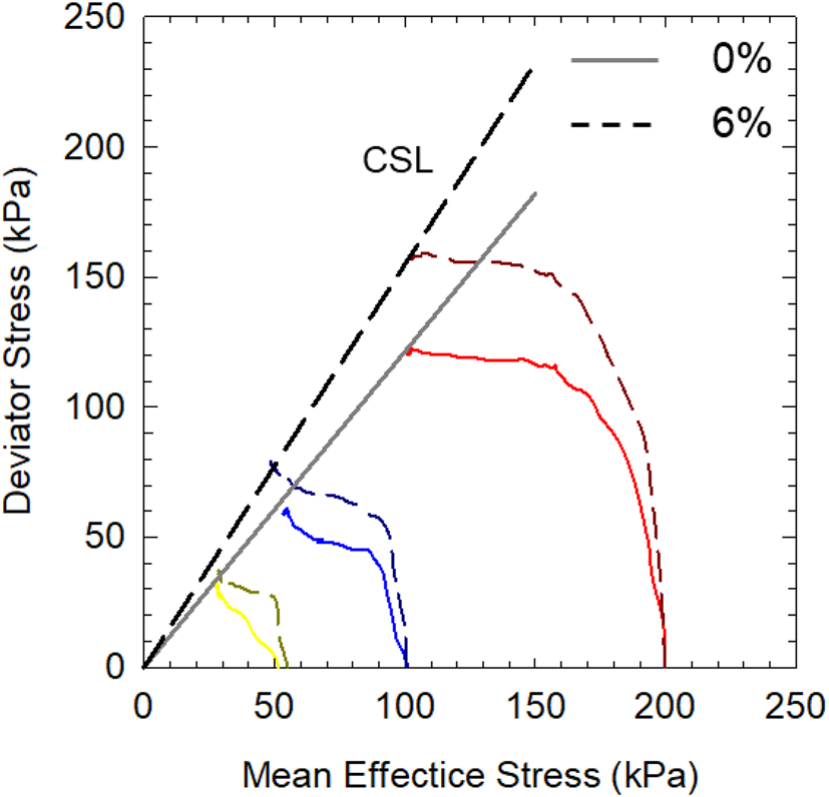
Comparison of effective stress path between soil specimens with DI water and 6% NaCl solution.

Figure 6 compares the effective stress paths of soil specimens with DI water and 6% NaCl solution at different mean effective stresses. The critical state lines show that the M value increases as the salinity increases from 1.20 with salinity at 0% (DI water) to 1.45 at 6% (NaCl), indicating that the stress path of non-saline soil has a larger deflection angle than that of saline soil. Wood^46^ proposed a relationship between the stress ratio at critical state M and internal friction angle φ′, as expressed in Eq. (5).

5 $$\mathrm{M}=\frac{6\mathrm{sin\varphi {^{\prime}}}}{3-\mathrm{sin\varphi {^{\prime}}}}$$

The M values of soil at different salinities were calculated by the above equation and compared with the measured M value shown in Table 5. It was found that the calculated values are highly approximate to the measured ones, suggesting the M value of soft saline clay in this study is a function of effective internal friction angle; thus, the CSL of soft marine clay at different salinities could be predicted by the effective internal friction angle.

**Table 5:** Comparison between calculated and measured stress ratio.

Salinity (%)	0	2	4	6
Calculated M	1.15	1.33	1.39	1.43
Measured M	1.17	1.34	1.39	1.45

**Figure 7:**
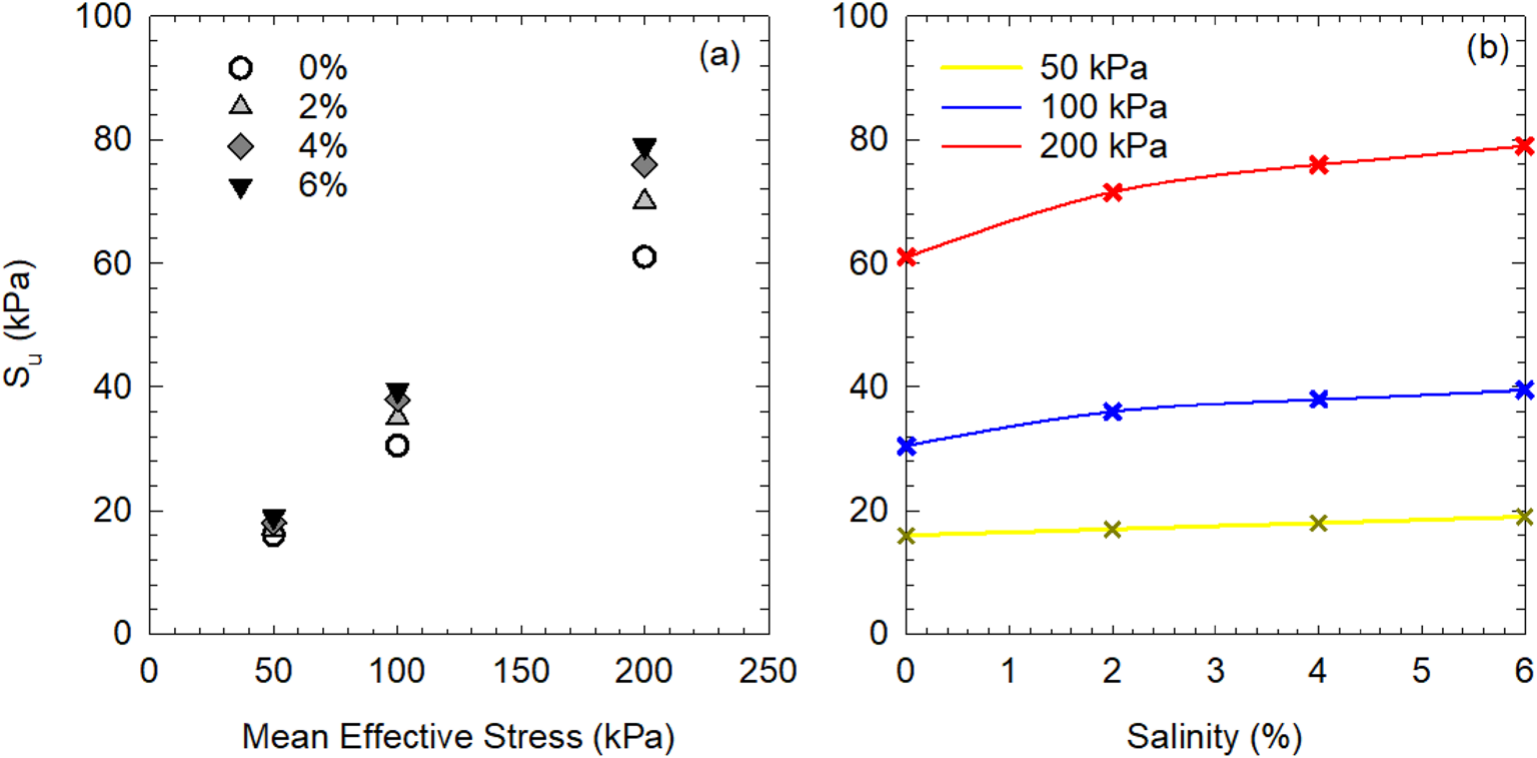
Undrained shear strength of soft marine soil versus (**a**) mean effective stress with different salinity, and (**b**) porewater salinity under different mean effective stress.

The undrained shear strength S_u_ from the consolidated undrained triaxial test was plotted with the mean effective stress p′, as shown in Fig. 7a. The S_u_ was taken as half of the maximum deviator stress (${\mathrm{q}}_{\mathrm{max}}/2$) in the case where the peak value was observed before reaching 15% axial strain, or half of the deviator stress at the axial strain of 15% in the case where no peak value was exhibited. The undrained shear strength increases with increasing mean effective stress for soil at all salinities, which fits the results of remolded clay presented by Yin et al.^47^. However, the increasing rate of undrained shear strength was different for soils at different salinities, where S_u_ of high salinity is higher than that of lower salinity under a given mean effective stress. It suggests that the porewater salinity would significantly affect the undrained shear strength of soft marine clay. Figure 7b shows the undrained shear strength versus porewater salinity under different mean effective stress. The undrained shear strength has an overall increasing trend with porewater salinity regardless of mean effective stress. Under low consolidation pressure (50 kPa), the increase in strength was relatively small (e.g., 3 kPa with the soil specimen of salinity from 0% NaCl to 6% NaCl); whereas the strength was significantly increased under high consolidation pressure (200 kPa), where S_u_ increased 30% from 61 to 79 kPa with the soil of salinity from 0 to 6%. It could attribute to the increased internal friction angle as large soil aggregates were formed. And the increasing rate in undrained shear strength was slowed as the porewater salinity kept increasing, where the aggregated and flocculated associations between soil particles were no longer evident (Fig. 4).

**Figure 8:**
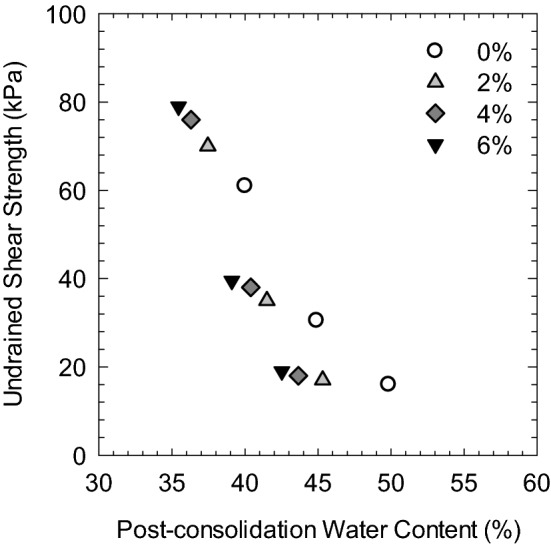
Undrained shear strength versus water content of soft marine clay at different salinities.

Figure 8 shows the relationship between undrained shear strength and the post-consolidation water content of soil specimens at different porewater salinities. The water content remained constant during shear and was of the same value as after consolidation since the test was conducted in undrained conditions. The undrained shear strength decreases as the water content increases regardless of porewater salinity, where the water content after shear was closely related to the initial water content of the soil. The undrained shear strength behavior can be explained in that the initial water content was low as the liquid limit of soil was reduced due to the effect of salt, in which condition the measured water content would be low after consolidation under a given confining pressure. Therefore, high undrained shear strength of soil was observed.

**Figure 9:**
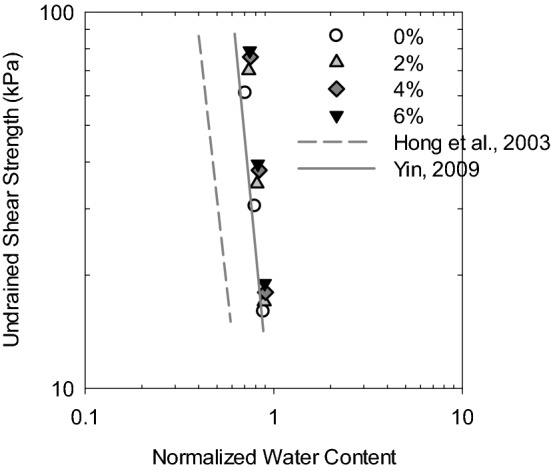
Relationship between consolidated undrained shear strength and normalized water content of soft marine clay with different salinities.

Hong et al.^48^ summarized the unconsolidated undrained shear strength of 115 remolded clays with a liquid limit lower than 150% all over the world and proposed a relationship between undrained shear strength and the normalized water content by a well-fitted empirically equation as expressed in Eq. (6).

6 $${\mathrm{C}}_{\mathrm{ur}}=1.4\times ({w{^{\prime}})}^{-4.5}$$

where C_ur_ is the undrained shear strength of remolded clay, and $\mathrm{w{^{\prime}}}$ is the normalized water content, defined as the ratio of natural water content (*w*) to the liquid limit (LL) of soil. Yin et al.^47^ normalized the water content after the consolidation of remolded clay and proposed a relationship between the triaxial consolidated undrained shear strength and the normalized water content described in Eq. (7).

7 $${\mathrm{S}}_{\mathrm{u}}=7.3\times {(w/\mathrm{LL})}^{-5.2}$$

where S_u_ is the undrained shear strength, *w* is the water content measured after consolidation, and LL is the liquid limit of soil. Figure 9 plots the undrained shear strength versus normalized water content of soil specimens at different porewater salinities in this study along with the empirical equations proposed by Hong et al.^48^ and Yin et al.^47^. It was found that the consolidated undrained shear strength S_u_ was overall higher than the unconsolidated undrained strength C_ur_ for given normalized water content due to the consolidation before shearing. The data set obtained in this study is well fitted with the relationship proposed by Yin et al.^47^ in a double logarithm scale, especially for the soil specimens with DI water (0% NaCl). However, the undrained shear strength increases as the porewater salinity increases, suggesting that porewater chemistry would dominate the consolidation undrained behavior of soft marine clay at a given water content. Note that LL would be altered by the porewater salinity of soil due to the osmotic effect in montmorillonite; thus, the actual normalized water content needs to be corrected by the intrinsic liquid limit of soil at each salinity in exhibiting the relationship with undrained shear strength.

**Figure 10:**
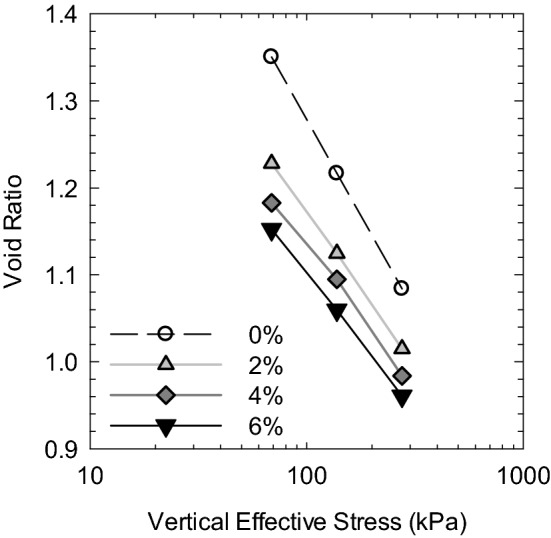
Triaxial compression curves of soft marine clay with different salinities.

Chandler^49^ proposed the concept of intrinsic S_u_ line (IS_u_L) for remolded clay by considering the void index, I_v_^50^, where the undrained shear strength of remolded clay can be expressed in terms of vertical effective stress in K_0_ condition (Eq. 8):

8 $${\mathrm{S}}_{\mathrm{u}}=0.33{\upsigma }_{\mathrm{v}}^{{{\prime}}}$$

where ${\upsigma }_{\mathrm{v}}^{{{\prime}}}$ is the vertical consolidation pressure on the intrinsic compression line (ICL). Since the ICL is based on the one-dimensional compression test with remolded clays, where there is no lateral deformation (e.g., K_0_ consolidation). There is a need to convert the consolidation curve of isotopic consolidation in the triaxial test to that of one-dimensional compression test, which could be achieved by using Eq. (9).

9 $${\mathrm{p}}^{{{\prime}}}=\frac{{\upsigma }_{\mathrm{v}}^{{{\prime}}}+2{\upsigma }_{\mathrm{h}}^{{{\prime}}}}{3}=\frac{{\upsigma }_{1}^{{{\prime}}}+2{\upsigma }_{3}^{{{\prime}}}}{3}=\frac{{\upsigma }_{\mathrm{v}}^{{{\prime}}}+2{\mathrm{K}}_{0}{\upsigma }_{\mathrm{v}}^{{{\prime}}}}{3}$$

where ${\upsigma }_{\mathrm{v}}^{{{\prime}}}$ is the vertical effective stress, ${\upsigma }_{\mathrm{h}}^{{{\prime}}}$ is the horizontal effective stress, ${\upsigma }_{1}^{{{\prime}}}$ and ${\upsigma }_{3}^{{{\prime}}}$ are the major and minor effective stresses, respectively, and K_0_ is the coefficient of earth pressure at rest defined by the ratio of horizontal and vertical effective stresses. Figure 10 shows the *e* ~ log*σ'*_*v*_ compression curves of equivalent confining pressure with soils at different salinities converted from the above-discussed method. The saturation was considered 100% since soil specimens were back pressure saturated. The void ratio was obtained by multiplying the measured water content after consolidation with the specific gravity of soil ($\mathrm{e}=\mathrm{w}\times {\mathrm{G}}_{\mathrm{s}}$). The compression curve of remolded clay at high salinity locates below that at relatively lower salinity, indicating a decreased compressibility of soft marine clay under the increased concentration of the saline solution. As the salinity keeps increasing, the decreasing rate of compressibility slows down. Meanwhile, the difference in compression curves reduces as the consolidation pressure increases with soil at all salinities, suggesting that higher consolidation pressure can eliminate the effect of salinity on the soil.

Burland^50^ proposed the void index, I_v_, for normalizing the compression behavior of remolded soil with initial water content at 1.25 times the liquid limit, as expressed in Eq. (10).

10 $${\mathrm{I}}_{\mathrm{v}}=\frac{\mathrm{e}-{\mathrm{e}}_{100}^{*}}{{\mathrm{e}}_{100}^{*}-{\mathrm{e}}_{1000}^{*}}=\frac{\mathrm{e}-{\mathrm{e}}_{100}^{*}}{{\mathrm{C}}_{\mathrm{c}}^{*}}$$

where the ${\mathrm{e}}_{100}^{*}$ and ${\mathrm{e}}_{1000}^{*}$ are the void ratio of remolded clay at the applied stress of 100 kPa and 1000 kPa, respectively. ${\mathrm{C}}_{\mathrm{c}}^{*}$ is the intrinsic compression index (${\mathrm{e}}_{100}^{*}-{\mathrm{e}}_{1000}^{*}$). Then the intrinsic compression line (ICL) can be expressed in terms of the vertical effective stress (σ_v_^*^) as follows:

11 $${\mathrm{I}}_{\mathrm{v}}=2.45-1.285{\mathrm{log\sigma }}_{\mathrm{v}}^{{{\prime}}}+0.015 {({\mathrm{log\sigma }}_{\mathrm{v}}^{{{\prime}}})}^{3}$$

**Figure 11:**
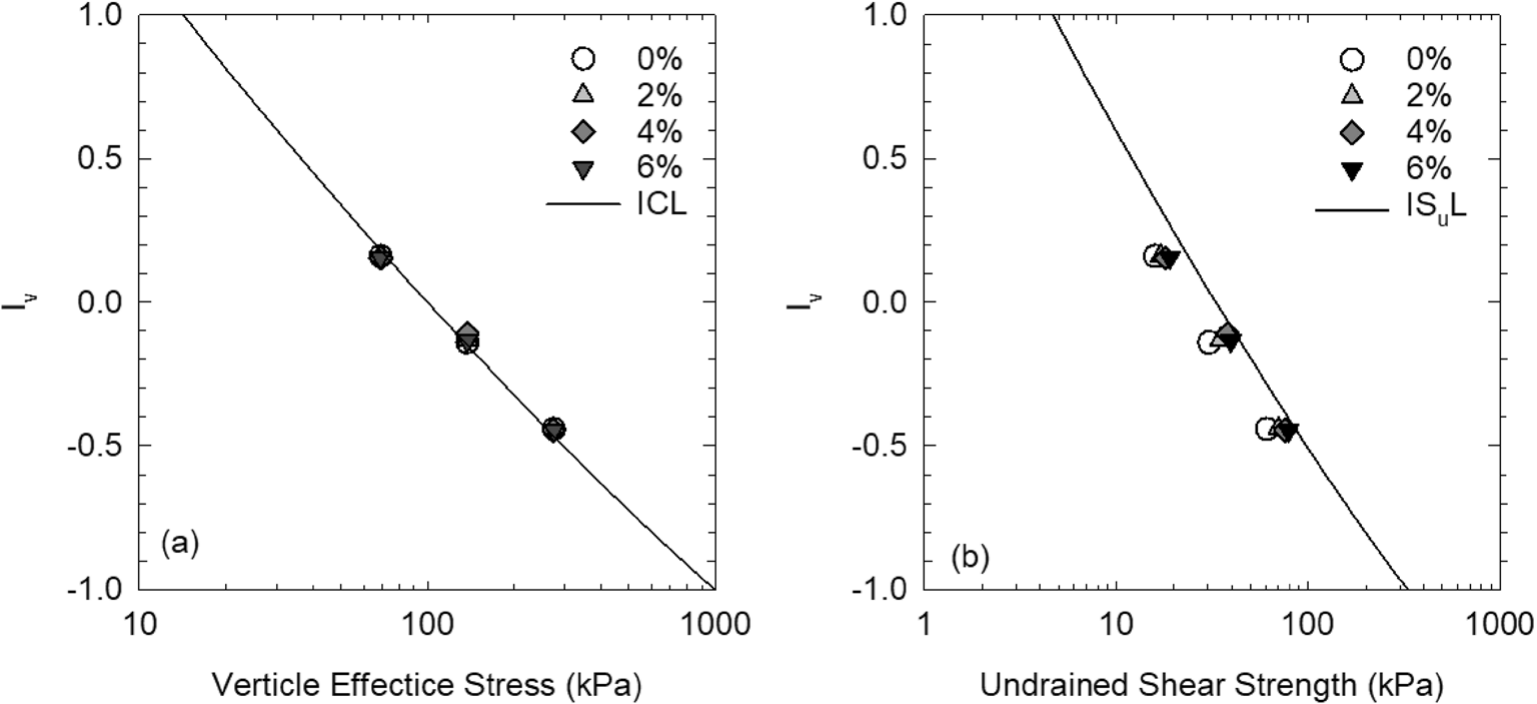
Void index versus (**a**) vertical effective pressure, and (**b**) undrained shear strength of soft marine clay with different porewater salinity.

Figure 11a plots the void index as a function of vertical effective stress of soils at different salinities. The compression lines of soil well fit the ICL line regardless of porewater salinity. Figure 11b shows the void index versus the undrained shear strength with IS_u_L. The results show that the undrained shear strengths of soft marine clay at different porewater salinities are close to the IS_u_L, especially with the soil at higher porewater salinity (e.g., 6%) since S_u_ was enhanced with the saline solutions.

Based on the above observations, particular emphasis needs to be put on the effect of salinity on the undrained strength of soil for engineers to have a conservative design when dealing with soft marine clays in the field, since environmental conditions (i.e., extreme rainfall) would temporarily decrease the soil salinity and exhibit the *short-term* results (e.g., decreased internal friction angle, reduced undrained shear strength).

### Analysis of the structure of soft marine clay

#### Imaging analysis of soft marine clay

**Figure 12:**
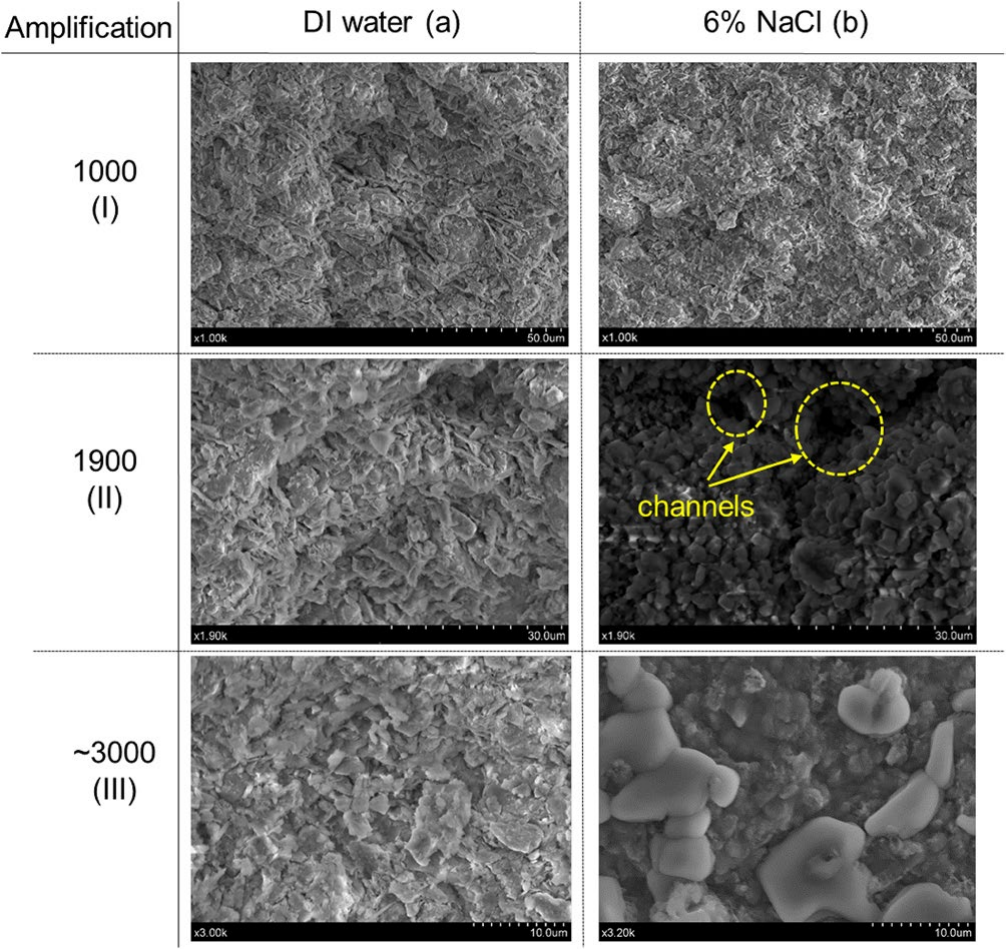
Upscaling SEM images of soft marine clay exposed to (**a**) DI water and (**b**) 6% NaCl solution.

Soil is composed of particles and voids, and the soil properties, i.e., compressibility, hydraulic conductivity, strength, etc., depending on particle size, shape, and/or arrangement^51^. Scanning electron microscopy (SEM) analysis was conducted on the soil to develop the morphology characteristics of soft marine clay under the effect of saline solutions. Figure 12 shows a sequence of SEM images at different scales with soil specimens exposed to DI water and 6% NaCl, respectively. Soil samples were back pressure saturated and applied with a confining pressure of 100 kPa prior to SEM specimen preparation. The flake and honeycomb structures are observed in the image of soil exposed to DI water, which is the typical soil morphology of illite. The complementary SEM images support the result obtained from the XRD analysis. The soil fabric changes from the flake structure (Fig. 12a) to the aggregated structure (Fig. 12b) as the salinity increases from 0 to 6%. The pore structure could not be clearly observed for the images amplified by 1000 times (Fig. 12I), where relatively compacted soil fabric was presented with soils exposed to both DI water and 6% NaCl. In the scaled-up images (e.g., amplified by 1900 and 3000 times), the particle aggregation with the saline solution could be more clearly observed, where the particle orientation and pore size distribution were exhibited to some extent (Fig. 12bII and bIII). Soil fabric exposed to DI water is relatively dispersed with no clear particle orientation (Fig. 12a). Conversely, soil fabric exposed to 6% NaCl solution presents the aggregated structure, contributing to increased internal friction angle and enhanced shear strength. Soil fabric changes from edge-to-edge and/or edge-to-face dominant association to face-to-face and edge-to-face dominant association as the porewater salinity increases from 0% (DI water) to 6% (NaCl), as indicated conceptually in Fig. 4. Though the pores are irregularly distributed, no large void was observed with soil exposed to DI water (Fig. 12a). However, there are relatively large channels observed with soil exposed to 6% NaCl, where the pores are connected through aggregated soil particles (Fig. 12bII). This pore morphology allows the soil to be more permeable. Salt crystal was observed on the surface of aggregated soil particles in the image amplified 3000 times (Fig. 12bIII), whereas flakes were presented in the image of soil exposed to DI water (Fig. 12aIII). The SEM images well exhibit the soil fabric of soft marine clay exposed to DI water and at 6% NaCl.

#### Quantitative analysis of the structure of soft marine clay

**Figure 13:**
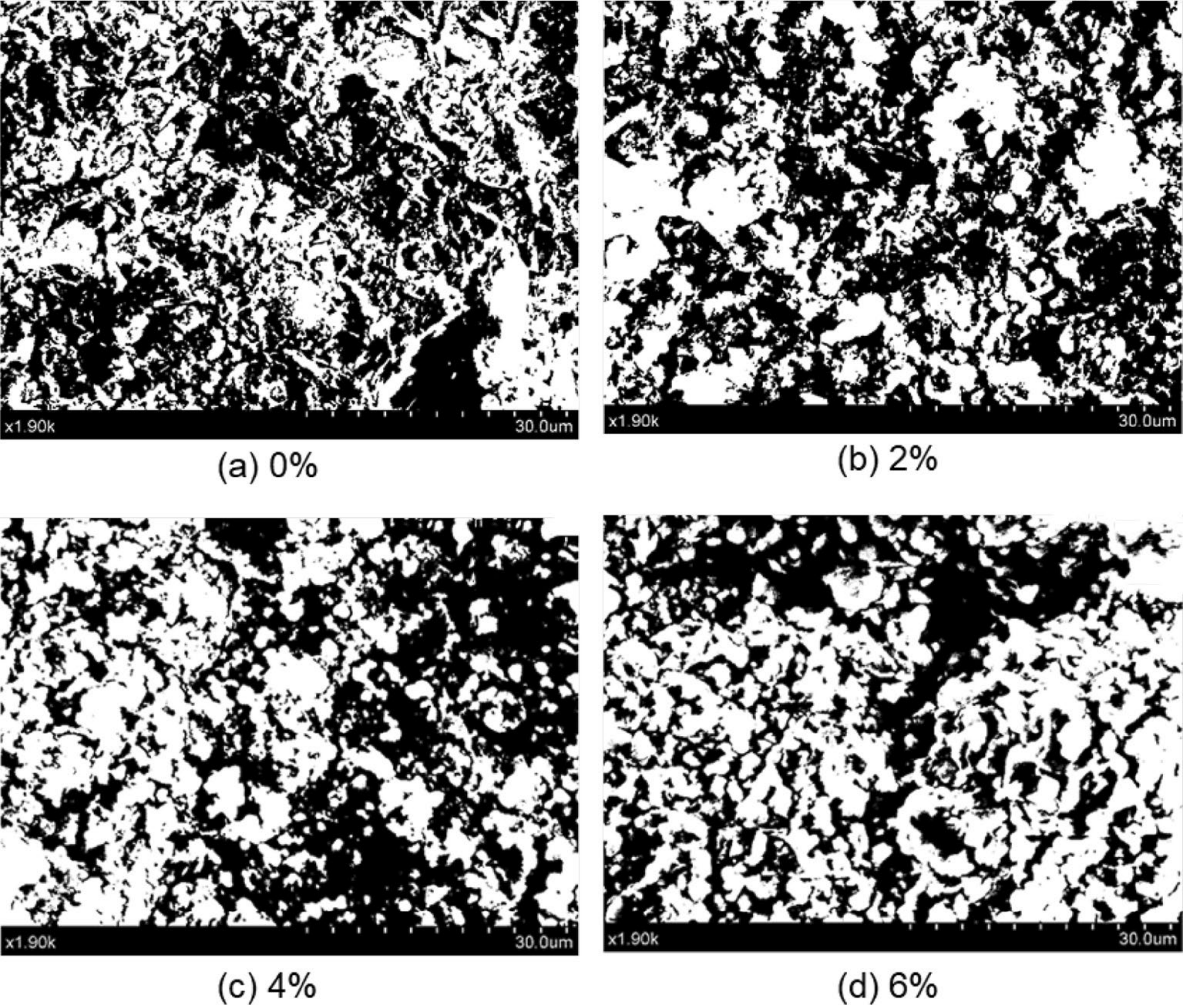
Binary images of soft marine clay with different salinities.

The pore structure of saline soil alters the mechanical behaviors of soil^52,53^. Al-Bared et al.^54^ summarized the physical properties of marine clay from several sites and stated that the micro-structure arrangement was the main factor affecting the strength of marine soil. The pore structures presented in terms of morphological indexes of soil at different salinities were anticipated to relate to soil strength in this study, and served as the potential input variables to predict the strength behaviors of marine clay. Images obtained from SEM analysis were imported to the Image-pro plus (IPP) software to investigate particle morphology quantitatively. The maximum entropy principle was employed for processing soil images at different salinities. As shown in Fig. 13, more aggregations (white region) are observed visually with increasing porewater. Information on the soil particles (e.g., area, roundness) and pores (e.g., area, orientation, and fractal dimension) were extracted from the binary images using the *count and measure* function of the IPP program and summarized in Table 6. The average value of selected areas for each image is taken herein for improved accuracy.

**Table 6:** Microscopic morphological parameters of soft marine clay with different salinities.

Salinity	Area of particles	Roundness of particles	Area of pores	Pore orientation	Fractal dimension of pore distribution
%	%	–	%	–	–
0	56.63	0.48	43.37	0.42	1.27
2	59.75	0.52	40.25	0.38	1.24
4	62.54	0.53	37.46	0.36	1.21
6	64.22	0.55	35.78	0.33	1.19

**Figure 14:**
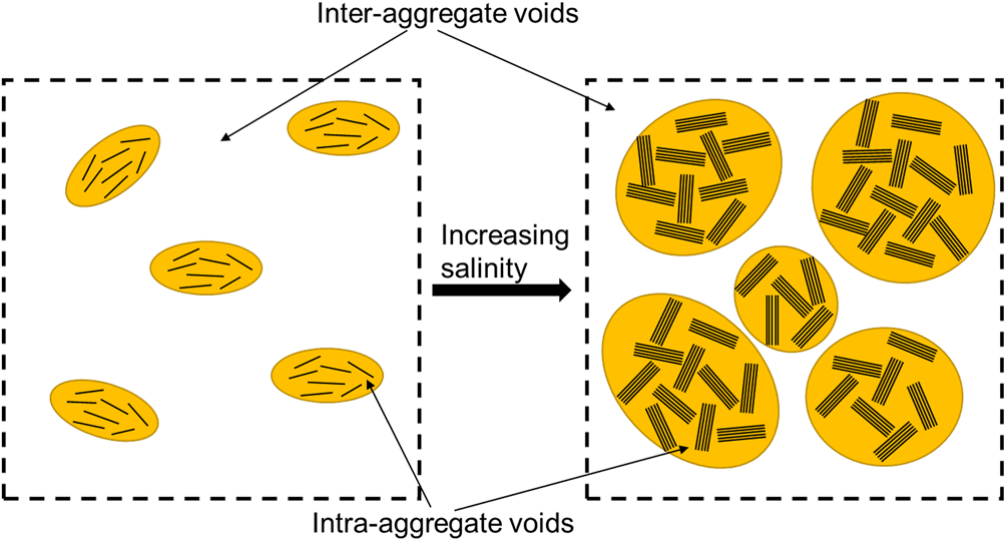
Conceptual representation unit of soft marine clay.

As the porewater salinity increases from 0 to 6%, the area of soil particles increases from 56.63 to 64.22%; meanwhile, the area of pores decreases by approximately 7.59%. Under the effect of saline solution, soil particles start to aggregate, in which the volume of large pores (intra-aggregate pores) was reduced since they were divided into fractions and smaller pores (interlayer pores and/or inter-aggregate pores). As the salinity increases, a flocculated and aggregated association between soil pellets (Fig. 4), composed of an aggregated soil structure. Thus, for a soil unit of constant volume, the area of soil particles increases and the total pore volume (measured primarily as the inter-aggregate pores) decreases as a result of particle aggregation, conceptually presented in Fig. 14. This framework fits the results from compression tests^47^, where a smaller reduction in voids was observed with increasing porewater salinity. It needs to point out that some soil particles entrapped in large intra-aggregate pores were considered part of pores due to the roughness of the soil surface and brightness of images. Therefore, the pore fraction extracted by the program is larger than the actual value, where there is a discrepancy between the void ratio obtained by IPP and that measured by the conventional soil mechanical method. Soil roundness is defined as the ratio of the average radius of curvature of the corners and edges of the particle to the radius of the maximum sphere that can be inscribed^55^. The value varies from 0 to 1, where the up end indicates a well-rounded particle. The roundness of soil particles increases slightly with increasing porewater salinity due to the aggregation effect.

Pore orientation is the frequency of pores in each azimuth angler section, which indicates the directional arrangement of soil pores. Generally, pores are more evenly arranged with a lower pore orientation value. The aggregation of soil particles makes the pores well aligned, decreasing pore orientation as the salinity increases. The results presented in this study fit the conclusion stated by Dor et al.^56^. The fractal dimension of pore distribution describes the distribution of soil pores projected on the plane in quantitative analysis of cohesive soils. A high value in the fractal dimension of pore distribution suggests poorly distributed pores, where the area of pores is more diverse as a result of less presented aggregations. The fractal dimension of pore distribution decreases with the increase of salinity, indicating that the distribution of pores develops towards a uniform trend as the soil particles aggregate.

### Linking structure features to the shear strength of soft marine clay

**Figure 15:**
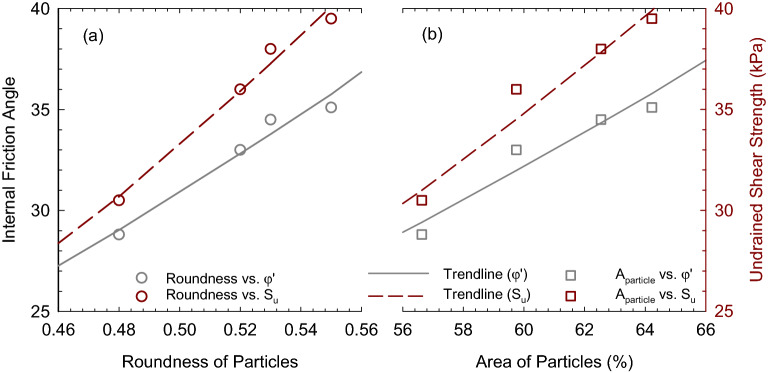
Internal friction angle and undrained shear strength versus particle index (**a**) roundness of particles, and (**b**) area of particles.

Detailed morphological analysis of soft marine clay at different porewater salinities suggests that the saline solution would make soil particles more aggregated. In this section, the influence of these morphologies on the macroscopic engineering behaviors of soft marine clay is evaluated. Figure 15 shows the internal friction angle (left axis) and undrained shear strength (right axis) as a function of soil particle index in (a) roundness of particles and (b) area of particles. The strength parameters generally increase with the increasing particle index, suggesting the presentation of aggregated soil structure. The roundness of particles is the degree of smoothing due to abrasion of soil particles, expressed as the ratio of the average radius of curvature of the edges and/or corners to the radius of curvature of the maximum inscribed sphere. More specifically, as the roundness of particles increases, soil particles tend to compact tightly, in which the formation of aggregation is favorable. The area of the particle measured by IPP is the area of aggregations since particles of smaller size could not be detected due to the limitation in the pixel of SEM images. Therefore, a large area of particles indicates a higher degree of aggregation in the soil, contributing to enhanced soil strength. Power functions were found well fit the relationships between undrained parameters and particle indexes, the fitting equations were summarized in Table 7 with the coefficient of determination. However, the data obtained in this study is limited; a more comprehensive data range is expected to better fit the relationships in the future.

**Table 7:** Summary of trendlines between undrained shear parameter and microscopic soil index.

Undrained parameters	${\mathrm{\varphi }}^{{\prime}}$	R^2^	S_u_	R^2^
Area of particles, A_particle_	$0.054{({\mathrm{A}}_{\mathrm{particles}})}^{1.56}$	0.92	$0.009{({\mathrm{A}}_{\mathrm{particles}})}^{2}$	0.95
Roundness of particles, R	$89.3{\mathrm{R}}^{1.53}$	0.96	$130{\mathrm{R}}^{1.97}$	0.98
Area of pores, A_pore_	$90\mathrm{exp}(- 0.026{\mathrm{A}}_{\mathrm{pores}})$	0.91	$131.7\mathrm{exp}(- 0.033{\mathrm{A}}_{\mathrm{pores}})$	0.94
Pore orientation, H	$75.9\mathrm{exp}(- 2.256\mathrm{H})$	0.90	$106.4\mathrm{exp}(- 2.292\mathrm{H})$	0.94
Fractal dimension of pore distribution, D_p_	$635.3\mathrm{exp}(- 2.42{\mathrm{D}}_{\mathrm{p}})$	0.89	$1653.2\mathrm{exp}(- 3.12{\mathrm{D}}_{\mathrm{p}})$	0.92

**Figure 16:**
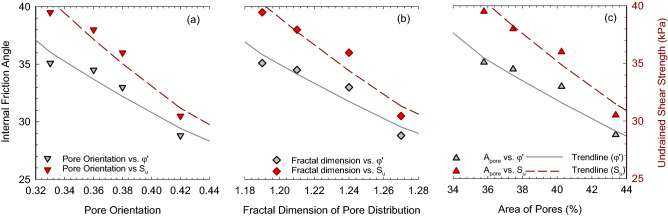
Internal friction angle and undrained shear strength versus pore index (**a**) pore orientation, (**b**) fractal dimension of pore distribution, and (**c**) area of pores.

Figure 16 shows the internal friction angle (left axis) and undrained shear strength (right axis) as a function of the void (pore) index in (a) pore orientation, (b) fractal dimension of pore distribution, and (c) area of pores. The strength parameters are observed to decrease with the increasing void index. As the pore orientation decreases, the pores in soil are better arranged, where soil particles are easier to compact. The compacted particles form large aggregates with a reduced area of pores and decreased fractal dimension of pore distribution, resulting in enhanced strength and reduced soil compressibility. In addition, the large pores forming the channels make the water flow easily through the soil, resulting in higher hydraulic conductivity with increasing porewater salinity^52^. Exponential functions were found well fitted in the relationships between undrained parameters and void indexes (Table 7). Similar to the relationships with particle index, a more comprehensive experimental data range with marine clays from other sites worldwide is expected to better fit the relationships in future studies.

## Conclusions and recommendations

The salinity effect on the strength behaviors of soft marine clay from Lianyungang Port was evaluated through a series of isotopically consolidated undrained triaxial tests. Soil at higher salinity was found to have increased strength and decreased compressibility. The undrained shear strength (S_u_) increases linearly with increasing mean effective stress. And S_u_ of soil at high salinity is higher than that of soil at relatively lower salinity, the difference in S_u_ enlarges as the confining pressure increases. S_u_ decreases with increasing post-consolidation water content and has a noticeable increase as the porewater salinity increases (0–6%). It scatters around the IS_u_L line by normalizing the water content, where that of soil at high salinity (6%) locates closest to the IS_u_L. The stress paths of soil at different salinities increase initially and then turn towards the up-left to the critical state. The stress ratio M increases with increasing salinity, where the CSL line could be determined by the empirical equation as a function of effective internal friction angle.

The enhanced strength behaviors could be attributed to the particle aggregation due to increased pore water salinity. The aggregated and flocculated associations were observed in the soil fabric as the porewater salinity increased, where the flake structure changed to the aggregated structure. The aggregation is hypothesized to increase the internal friction angle, resulting in enhanced undrained shear strength. Detailed morphology analyses show the relationships between macroscopic engineering properties and the microscopic particle and/or pore indexes. The internal friction angle and undrained shear strength increase with increasing roundness of particles and the area of particles, and decrease with increasing void (pore) indexes including pore orientation, the fractal dimension of pore distribution, and the area of pores. The quantitative relationships between strength behaviors and morphological indices could serve as potential screening tools for assessing the strength of in-situ marine clay in the Lianyungang region. Future study is recommended on marine clays of similar geological history from other sites worldwide to obtain a broader range of experimental data to better fit the empirical equations and to allow more accurate predictions in targeted engineer properties with known soil index.

## References

[1] Chung SG, Giao PH, Nagaraj TS, Kwag JM (2002). Characterization of estuarine marine clays for coastal reclamation in Pusan, Korea. Mar. Georesour. Geotechnol..

[2] Jung YH, Finno RJ, Cho W (2012). Stress–strain responses of reconstituted and natural compressible Chicago glacial clay. Eng. Geol..

[3] Karakouzian M, Avar BB, Hudyma N, Moss JA (2003). Field measurements of shear strength of an underconsolidated marine clay. Eng. Geol..

[4] Deng Y (2014). Effect of pore water chemistry on the hydro-mechanical behaviour of Lianyungang soft marine clay. Appl. Clay Sci..

[5] Bo MW, Arulrajah A, Sukmak P, Horpibulsuk S (2015). Mineralogy and geotechnical properties of Singapore marine clay at Changi. Soils Found..

[6] Anggraini V, Asadi A, Syamsir A, Huat BB (2017). Three point bending flexural strength of cement treated tropical marine soil reinforced by lime treated natural fiber. Measurement.

[7] Yin J, Han W, Xu G, Hu M, Miao Y (2019). Effect of salinity on strength behavior of cement-treated dredged clay at high initial water contents. KSCE J. Civ. Eng..

[8] Ahmad, N. R. & Harahap, I. S. The compression behaviour of marine clays in Malaysia. In *International Conference on Offshore Mechanics and Arctic Engineering, American Society of Mechanical Engineers*, vol. **49927**, V001T10A011. 10.1115/OMAE2016-54795 (2016).

[9] Arulrajah A, Nikraz H, Bo MW (2005). In-situ testing of Singapore marine clay at Changi. Geotech. Geol. Eng..

[10] Jiang N (2015). Multi-scale laboratory evaluation of the physical, mechanical, and microstructural properties of soft highway subgrade soil stabilized with calcium carbide residue. Can. Geotech. J..

[11] Zainuddin N (2019). Measuring the engineering properties of marine clay treated with disposed granite waste. Measurement.

[12] Church JA (2013). Sea Level Change.

[13] Locat A (2011). Progressive failures in eastern Canadian and Scandinavian sensitive clays. Can. Geotech. J..

[14] Nicholls RJ, Cazenave A (2010). Sea-level rise and its impact on coastal zones. Science.

[15] Rankka, K. *et al*. In *Quick clay in Sweden*. SGI Report No. 65. http://www.swedgeo.se (2004).

[16] Taylor M, Krüger N (2019). Changes in salinity of a clay soil after a short-term salt water flood event. Geoderma Reg..

[17] Ter-Stepanian G (2000). Quick clay landslides: Their enigmatic features and mechanism. Bull. Eng. Geol. Environ..

[18] Deng Y (2018). Pore water salinity effect on the intrinsic compression behaviour of artificial soft soils. Appl. Clay Sci..

[19] Horpibulsuk S, Yangsukkaseam N, Chinkulkijniwat A, Du Y (2011). Compressibility and permeability of Bangkok clay compared with kaolinite and bentonite. Appl. Clay Sci..

[20] Yukselen-Aksoy Y, Kaya A, Ören AH (2008). Seawater effect on consistency limits and compressibility characteristics of clays. Eng. Geol..

[21] De Rosa J, Pontolillo DM, Maio CD, Vassallo R (2016). Chemical clay soil improvement: From laboratory to field test. Procedia Eng..

[22] Di Maio C (1996). Exposure of bentonite to salt solution: Osmotic and mechanical effects. Géotechnique.

[23] He P, Ohtsubo M, Abe H, Higashi T, Kanayama M (2014). Quick clay development and cation composition of pore water in marine sediments from the Ariake Bay area, Japan. Int. J. Geosci..

[24] Mokni N, Romero E, Olivella S (2014). Chemo-hydro-mechanical behaviour of compacted Boom Clay: Joint effects of osmotic and matric suctions. Géotechnique.

[25] Siddiqua S, Blatz J, Siemens G (2011). Evaluation of the impact of pore fluid chemistry on the hydromechanical behaviour of clay-based sealing materials. Can. Geotech. J..

[26] Tiwari B, Ajmera B (2015). Reduction in fully softened shear strength of natural clays with NaCl leaching and its effect on slope stability. J. Geotech. Geoenviron. Eng..

[27] Zhang L, Sun D, Jia D (2016). Shear strength of GMZ07 bentonite and its mixture with sand saturated with saline solution. Appl. Clay Sci..

[28] Spagnoli G, Fernández-Steeger T, Feinendegen M, Azzam R, Stanjek H (2011). Influence of the dielectric constant, electrolyte concentration and pH of the pore fluids on the shear strength of monomineralic clays. Ital. Geotechn. J..

[29] Sridharan A, Prakash K (2000). Influence of clay mineralogy and pore-medium chemistry on clay sediment formation. Can. Geotech. J..

[30] Maio CD, Fenellif GB (1994). Residual strength of kaolin and bentonite: The influence of their constituent pore fluid. Géotechnique.

[31] Rajasekaran G, Rao SN (2004). Falling cone method to measure the strength of marine clays. Ocean Eng..

[32] Bushra, I. & Robinson, R. G. Consolidation behaviour of a cement stabilised marine soil. In *Proceedings of International Geotechnical Conference* 431–434 (2009).

[33] Xiao HW, Lee FH (2016). Curing time effect on behavior of cement treated marine clay. World Acad. Sci. Eng. Tech..

[34] Izabel KJ, Sangeetha S (2014). Stabilization of marine clay using jerofix. Int. J. Sci. Eng. Res..

[35] Prasad DSV, Venkatteswarlu H, Rao NJ, Kumar JC (2015). Strength behaviour of marine clay treated with rice husk ash. Int. J. Eng. Sci. Res. Tech..

[36] Al-Bared MAM, Marto A, Latifi N, Horpibulsuk S (2018). Sustainable improvement of marine clay using recycled blended tiles. Geotech. Geol. Eng..

[37] Al-Bared MAM, Mustaffa Z, Armaghani DJ, Marto A, Yunus NZM, Hasanipanah M (2021). Application of hybrid intelligent systems in predicting the unconfined compressive strength of clay material mixed with recycled additive. Transport. Geotechn..

[38] Al-Obaidi, A., Ihssan, A., Allawi, H., Al-Attar, T. S., Al-Neami, M. A. *et al*. Studying of the combined salts effect on the engineering properties of clayey soil. In *Matec Web of Conferences* 162. 10.1051/matecconf/201816201011 (2018).

[39] Yan WM, Chang J (2015). Effect of pore water salinity on the coefficient of earth pressure at rest and friction angle of three selected fine-grained materials. Eng. Geol..

[40] Sridharan A, El-Shafei A, Miura N (2002). Mechanisms controlling the undrained strength behavior of remolded ariake marine clays. Mar. Georesour. Geotechnol..

[41] Paassen LAV, Gareau LF (2004). Effect of pore fluid salinity on compressibility and shear strength development of clayey soils. Eng. Geol. Infrastruct. Plan. Europe.

[42] Wu Z, Deng Y, Cui Y, Chu C, Feng Q (2020). Geological investigation of the settlement behaviour of two highways in Lianyungang region. Eng. Geol..

[43] Sridharan A, Prakash K (1999). Mechanisms controlling the undrained shear strength behaviour of clays. Can. Geotech. J..

[44] van Paassen, L. A. & Gareau, L. F. Effect of pore fluid salinity on compressibility and shear strength development of clayey soils. In *Engineering Geology for Infrastructure Planning in Europe* 327–340 (Springer, Berlin, Heidelberg, 2004). 10.1007/978-3-540-39918-6_39.

[45] Yin J, Lu Z, Geng W, Han W, Hudu A (2021). Effect of porewater salinity on compression behaviors and hydraulic conductivity of soft marine clay. Mar. Georesour. Geotechnol..

[46] Wood DM (1991). Soil Behaviour and Critical State Soil Mechanics.

[47] Yin J, Gao Y, Hong Z (2009). Research on undrained shear strength tests of soft Lianyungang clay. Rock Soil Mech..

[48] Hong Z, Liu H, Takehito N (2003). Remolded undrained strength of soils. China Ocean Eng..

[49] Chandler RJ (2000). Clay sediments in depositional basin: The geotechnical cycle. J. Eng. Geol. Hydrol..

[50] Burland JB (1990). On the compressibility and shear strength of natural clays. Géotechnique.

[51] Mitchell JK, Soga K (2005). Fundamentals of Soil Behaviour.

[52] Geng, W. Assessing the performance of polymer-bentonite mixtures for hydraulic barrier applications. In *ProQuest; University of Wisconsin—Madison* (2018).

[53] Siddiqua S, Siemens G, Blatz J, Man A, Lim BF (2014). Influence of pore fluid chemistry on the mechanical properties of clay-based materials. Geotech. Geol. Eng..

[54] Al-Bared MAM, Marto A (2017). A review on the geotechnical and engineering characteristics of marine clay and the modern methods of improvements. Malays. J. Fundam. Appl. Sci..

[55] Wadell, H. Volume, shape and roundness of rock particles. *J. Geol.* **43**, 250–280. http://www.jstor.org/stable/30056250 (1935).

[56] Dor M, Levi-Kalisman Y, Day-Stirrat RJ, Mishael Y, Emmanuel S (2020). Assembly of clay mineral platelets, tactoids, and aggregates: Effect of mineral structure and solution salinity. J. Colloid Interface Sci..

